# Association of tumor immune microenvironment profiling and 21-gene recurrence assay in early breast cancer patients

**DOI:** 10.1186/s40001-022-00917-3

**Published:** 2022-12-17

**Authors:** Yiwei Tong, Jiahui Huang, Weili Ren, Jing Yu, Xu Zhang, Zheng Wang, Jin Hong, Weiqi Gao, Jiayi Wu, Min Ji, Kunwei Shen, Xiaosong Chen

**Affiliations:** 1 Department of General Surgery, Comprehensive Breast Health Center, Ruijin Hospital, Shanghai Jiao Tong University School of Medicine, 197 Ruijin Er Road, Shanghai, 200025 China; 2 Department of Breast Surgery, Shaoxing Shangyu People’s Hospital, Shaoxing, 312300 Zhejiang China; 3 Department of Breast, International Peace Maternity and Child Health Hospital, Shanghai Jiao Tong University School of Medicine, 910 Hengshan Road, Shanghai, 200030 China

**Keywords:** Breast cancer, Tumor immune microenvironment, Immune profiling, 21-gene recurrence score, Prognosis

## Abstract

**Introduction:**

Tumor immune microenvironment (TIME) plays a vital role in breast cancer development, treatment resistance, and prognosis. This study evaluates the association of TIME profiling and 21-gene recurrence score (RS) in early Luminal breast cancer patients.

**Methods:**

ER+ /HER2-, pN0 breast cancer patients with available RS results who received surgery between January 2009 and December 2013 were enrolled. TIME markers, including stromal tumor infiltrating lymphocytes (TILs), CD3, CD4, CD8, and tumor PD-L1 expression, were comprehensively analyzed. Association of TIME markers with RS, as well as their correlation with breast cancer-specific survival (BCSS) were tested.

**Results:**

Overall, 385 patients were included, of whom 341 (88.6%) had TILs ≤10%. TIME markers were positively but moderately correlated with each other (Spearman r 0.28–0.53, all *P* < 0.05). Continuous RS showed a weak correlation with continuous TILs, CD3, CD8, and PD-L1. Regarding single gene mRNA level in the 21-gene RS panel, higher expression of TIME markers was related to lower ER group genes expression, but higher proliferation and invasion group genes level. After a median follow-up of 91.67 (range 5.03–134.03) months, TILs (*P* = 0.049) and PD-L1 (*P* = 0.034) were inversely associated with BCSS.

**Conclusions:**

Breast cancer TIME markers, including TILs, CD3, CD4, CD8, and PD-L1, were correlated with 21-gene RS score. Lower expression of ER group genes, as well as higher expression of proliferation and invasion group genes were associated with a higher level of these TIME markers, warranting further exploration.

**Supplementary Information:**

The online version contains supplementary material available at 10.1186/s40001-022-00917-3.

## Main text

### Introduction

Hormone receptor (HR)-positive, human epidermal growth factor receptor 2 (HER2)-negative disease is the most frequent breast cancer subtype, accounting for 60–70% of all cases [1]. Over the past decades, genetic factors have been integrated on the basis of classic clinicopathologic variables, which provided important information regarding the treatment decision and survival prediction of these patients. Among various guideline-approved multigene panels, the 21-gene recurrence score (RS) is the most frequently applied in clinical practice, to predict chemotherapy benefits and clinical outcomes for HR-positive, HER2-negative patients [2–4].

In addition to tumor-intrinsic factors, the tumor immune microenvironment (TIME) has increasingly captured considerable attention for its role in the development and progression of cancers [5]. Immune infiltrate, a crucial part of the tumor microenvironment which is often assessed by tumor infiltrating lymphocytes (TILs), has been proven prognostic and predictive in many solid malignancies including breast cancer [6]. Till now, TILs assessment has reached the evidence level of 1b as a prognostic marker in triple negative breast cancer [7], and its routine practice has been endorsed by international guidelines for the selected population [8, 9]. Previous evidence showed that the prognostic effect of TILs was heterogeneous among molecular subtypes. For instance, Carbognin et al. suggested a favorable effect for patients with high stromal TILs in both neoadjuvant and adjuvant settings, but only limited to triple-negative and HER2-overexpressed diseases [10]. In the cohort of Denkert et al*.*, high TILs infiltration was associated with improved disease-free and overall survival in triple-negative patients, but shorter overall survival in HR-positive, HER2-negative tumors [11], indicating a crucial need to better profile the TIME status in luminal breast cancer.

Significant heterogeneity exists in TILs subpopulations. In breast cancer, TILs were primarily composed of CD8^+^ cytotoxic and CD4^+^ helper T cells [11]. CD3 has also been used to detect intratumoral lymphocytes in different solid tumors [12–15]. Meanwhile, the expression of immune checkpoint molecules on TILs, for example, PD-L1, is an important mechanism of tumor immune escape [16]. Therefore, immune profiles including CD3, CD4, CD8, and PD-L1 have been increasingly studied so as to gain a deeper understanding of the tumor microenvironment. Yet, the evidence was mostly focusing on triple-negative or HER2-positive breast cancer, while the effect of different immune cell subsets remains complex and controversial. Currently, data were limited regarding the role of TIME profiling for luminal breast cancer patients. Moreover, the association between TIME profiling and multigene assay such as 21-gene RS has not been thoroughly elucidated.

In this study, we aimed to investigate the correlation between different TIME markers and to identify clinicopathologic features impacting TIME profiles in early luminal breast cancer patients. Moreover, we intended to explore the association of TIME markers with 21-gene RS, as well as their prognostic value.

### Materials and methods

#### Study population

Consecutive breast cancer patients who received breast surgery in Comprehensive Breast Health Center, Ruijin Hospital, Shanghai Jiao Tong University School of Medicine, Shanghai, China, between January 2009 to December 2013 were retrospectively reviewed. Those who met the following criteria were included: (a) female gender; (b) histologically proven invasive breast cancer; (c) ER or PR ≥ 1%, HER2 immunohistochemistry 0–1 + , or 2 + with fluorescence in situ hybridization-negative; (d) node-negative disease; (e) 21-gene RS result with available cycle threshold (*C*_T_) values for each gene; (f) complete follow-up information. Patients receiving neoadjuvant systemic treatment were excluded (Additional file 2: Figure S1). All participants gave informed consent for the use of tumor tissue and anonymous clinical data for research purposes. The current study was reviewed and approved by the independent Ethical Committees of Ruijin Hospital, Shanghai Jiao Tong University School of Medicine.

#### Data collection

Tumor histologic pathologic examination was conducted in the Department of Pathology, Ruijin Hospital by at least two independent, experienced pathologists (*X Fei, J Xie and X Jin*). The immunohistochemical (IHC) evaluation of estrogen receptor (ER), progesterone receptor (PR), HER2 and Ki-67 was as described in previous studies [17], based on the latest ASCO/CAP guidelines [18]. The cutoff for ER-high was set at $\ge$50% based on the St. Gallen International Expert Consensus on the Primary Therapy of Early Breast Cancer 2009 [19]. Tumors were classified into Luminal A-like (ER $\ge$1%/PR ≥ 20%/Ki-67 < 14%), and Luminal B-like (ER  ≥ 1%/PR < 20%/any Ki-67, ER or PR ≥ 1%/Ki-67 ≥ 14%) with respect to the 2013 St. Gallen Consensus [20].

Clinical data of participants were retrieved from Shanghai Jiao Tong University Breast Cancer Database (SJTU-BCDB). Follow-up was completed by specialized breast cancer nurses in our center. The clinical outcome of participants was measured according to the STEEP criteria, in terms of breast cancer-free interval (BCFI), which was calculated from the date of surgery to the recurrence of tumor including ipsilateral, local/regional or distant recurrence, and death from breast cancer. Another endpoint was breast cancer-specific survival (BCSS), which was calculated from the date of surgery till death from breast cancer [21]. Last follow-up was completed by July 2020.

#### 21-gene RS evaluation

The center-specific 21-gene RS assay was accomplished in the Department of Clinical Laboratory, Ruijin Hospital, by *L Lin* and *J Lin*, as described in our previous work [22, 23] (see Additional file 1). Following RNA extraction (RNeasy FFPE RNA kit; Qiagen, 73504, Germany), and reverse transcription (Omniscript RT kit; Qiagen, 205111, Germany), quantitative polymerase chain reaction (PCR) was conducted in Applied Biosystems 7500 Real-Time PCR System (Foster City, CA) using Premix Ex TaqTM (TaKaRa Bio, RR390A). Gene expression was measured by C_T_ value, which was verified in triplicate, and then normalized to five reference genes. The five housekeeping genes including $\beta$ *-actin*, *GAPDH*, *GUS*, *RPLPO*, and *TFRC* expressed at relatively constant rates under both normal and patho-physiological conditions, and were thus selected as reference genes. Relative gene expression was presented in form of $-\Delta$*C*_T_ $=$*C*_T Gene_ -*C*_T Reference_. RS was calculated according to the reference gene-normalized formula, and the cutoff of 25 was applied for this node-negative study population [2]. Our center-specific RS panel has been retrospectively validated in both node-negative and node-positive patients with two large cohorts of Chinese patients [22, 23], and has been routinely applied in selective breast cancer patients treated in our center to guide adjuvant treatment and predict disease outcome since 2015.

#### Quantification of TIME profiling

Sections of surgical specimens were stained and scanned according to the manufacturer's protocols. Counting of stromal TILs, stromal CD3, CD4, CD8, as well as PD-L1 were accomplished on digitalized images.

Stromal TILs were evaluated retrospectively according to the guidelines of the International TIL Working Group [24]. TILs were measured as the percentage of stromal immune cells infiltration within tumor borders. In case of heterogeneity, hot-spot and cold-spot regions were avoided and the average was reported. TILs counts were then categorized into TILs low (≤ 10%) and high (> 10%) groups.

The IHC assessment of stromal CD3, CD4, and CD8 was conducted with respect to the recommendations from the International Immunooncology Biomarkers Working Group [6]. Dewaxed sections were heated with Citrate Antigen Retrieval solution for antigen retrieval, incubated overnight at 4 °C with primary antibodies anti-CD3 (Abcam, ab16669, 1:100), anti-CD4 (Abcam, ab133616, 1:300), anti-CD8 (Abcam, ab101500, 1:100), and detected using IHC detection kit DAB. The median counts of CD3^+^ (5 cell count/mm^2^), CD4^+^ (1 cell count/mm^2^), and CD8^+^ (6 cell count/mm^2^) cells were applied as cutoffs for data analysis.

Tumors were further divided into three subgroups, namely, “immune inflamed”, “immune excluded”, and “immune desert” according to the spatial distribution of CD8^+^ cells. Immune inflamed is featured by comparable high frequencies of CD8 + T cells at tumor border and center. Immune excluded is defined as > 10 times more CD8^+^ cells at tumor border compared to center. Immune desert refers to hardly any CD8^+^ cells at both the border and center [25].

Tumor PD-L1 expression was tested using primary antibody anti-PD-L1 (Abcam, ab205921, clone 28–8, at 1:30 dilution; 1 h 37 °C) following the manufacturer's protocol. PD-L1 was evaluated by combined positive score (CPS), which was defined as the number of tumor cells, lymphocytes, and macrophages with positive PD-L1 membranous staining of any intensity in relation to total tumor cells. PD-L1 was considered positive if CPS ≥ 1%.

The enumeration of TIME markers was performed manually by three independent observers (*TILs: J Huang, J Hong, W Gao; CD3, CD4, CD8, PD-L1: Y Tong, Xu Zhang, Z Wang*) after satisfactory internal equity verification, and a two-round data proof quality control.

#### Statistical analysis

The relationship between clinicopathologic features and tumor immune profiles was investigated by univariate Chi-square test and multivariate binary logistic regression reporting odds ratio (OR) with 95% confidence interval (CI). Spearman’s rank correlation matrix was applied to detect the association between different TIME markers, and between continuous RS with TIME markers. Kruskal–Wallis test was adopted to compare the distribution of RS gene expression by different immune profiling statuses. Clinical outcomes were compared in form of Kaplan–Meier curves. Subgroup analysis was applied to test interaction between TIME markers and RS on survival. IBM SPSS version 25 (SPSS, Inc., Chicago, IL) and GraphPad Prism version 8.0 (GraphPad Software, CA, USA) were applied for statistical analysis and image construction. A two-sided *P* value < 0.05 was considered statistically significant.

### Results

#### Baseline characteristics

Overall, data from 385 Luminal-like, HER2-negative, node-negative patients were finally analyzed. Baseline clinicopathologic features are shown in Table 1. Among the included population, the median age was 56 (range 24–93) years. Invasive ductal carcinoma (IDC) was diagnosed in 84.2% of patients, and 72.5% had T1 tumors. Luminal A and B-like tumors consisted of 29.1% and 70.9% of the population. The median 21-gene RS was 30 (range 0–100), with 30, 135, 36, 184 patients carrying RS < 11, 11–25, 26–30, ≥ 31, respectively. Adjuvant treatment information was presented in Additional file 3: Table S1.

**Table 1:** Baseline characteristics

Characteristics	N	*%*
Age, years		
≤ 50	127	33.0
> 50	258	67.0
Menstrual status		
Premenopausal	144	37.4
Postmenopausal	241	62.6
Histologic type		
IDC	324	84.2
Non-IDC	61	15.8
Tumor size, cm		
** ≤ **2	279	72.5
> 2	106	27.5
Histological grade		
I	47	12.2
II	234	60.8
III	96	24.9
Unknown	8	2.1
ER expression, %		
< 50	79	20.5
≥ 50	306	79.5
PR status		
Positive	333	86.5
Negative	52	13.5
Ki-67 index, %		
< 14	189	49.1
≥ 14	196	50.9
Luminal subtype		
Luminal-A-like	112	29.1
Luminal-B-like	273	70.9
Recurrence score		
≤ 25	165	42.9
> 25	220	57.1

*IDC* invasive ductal carcinoma, *ER* estrogen receptor, *PR* progesterone receptor

#### Association of TIME profiling and clinicopathological factors

**Fig. 1:**
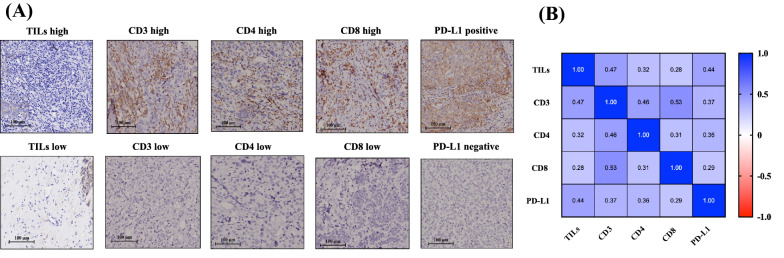
TIME markers. **A** Representative images of TILs and immunohistochemical staining of CD3, CD4, CD8, and PD-L1. **B** Correlation between TIME markers. Spearman’s rank correlation matrix was applied to detect the association between different TIME markers. *TIL* tumor infiltrating lymphocyte, *PD-L1* programmed cell death-ligand 1, *TIME* tumor immune microenvironment

With regard to the TIME markers, 341 (88.6%) patients had low TILs. A higher expression of CD3, CD4, and CD8 was found in 200, 152, and 200 patients, respectively. PD-L1-positive disease was found in 43 (11.2%) patients. The representative images for TILs and TIME marker staining are presented in Fig. 1A.

Clinicopathologic impact factors for each TIME marker were identified through univariate (Additional file 3: Table S2) and multivariate binary logistic regression (Additional file 3: Tables S3–S6). Higher TILs level was independently associated with higher tumor grade (II vs III, OR 0.39, 95% CI 0.18–0.82, *P* = 0.013), and Ki67 ≥ 14% (vs < 14%, OR 8.57, 95% CI 1.75–41.93, *P* = 0.008). Meantime, each TIME marker was associated with different clinicopathologic features: CD3 was significantly lower in IDC cases (OR 0.43, 95% CI 0.22–0.83, *P* = 0.012); CD4 was positively correlated with tumor size (≥  2 cm vs < 2 cm, OR 1.79, 95% CI 1.11–2.88, *P* = 0.017) and grade (I vs III, OR 0.40, 95% CI 0.16–0.99, *P* = 0.047); premenopausal (*vs* postmenopausal, OR 1.59, 95% CI 1.05–2.44, *P* = 0.028) and RS > 25 (vs ≤ 25, OR 1.66, 95% CI 1.10–2.50, *P* = 0.016) were independent predictors for higher CD8. When compared to the immune desert phenotype, immune inflamed tumors were more likely to have higher Ki67 (*P* = 0.039), higher RS (*P* = 0.023), and a tendency toward younger age (*P* = 0.061). Immune excluded cases shared more similarity with immune desert phenotype, except that the former was more often found in younger patients (*P* = 0.029). In addition, PR-negative tumors were more likely to have positive PD-L1 (OR 2.80, 95% CI 1.20–6.57, *P* = 0.029).

#### Correlation between TIME markers

The correlation test demonstrated that stromal TILs infiltration was positively associated with the expression of CD3, CD4, CD8, and PD-L1 (all *P* < 0.001; Fig. 1B) but the correlation was rather moderate (Spearman r 0.28–0.47). The greatest correlation was found between TILs with CD3 (Spearman r 0.47, *P* < 0.001), followed by TILs with PD-L1 (Spearman r 0.44, *P* < 0.001). All other TIME markers were positively correlated with each other (all *P* < 0.05), with Spearman’s *r* value 0.29–0.53. CD3 showed a fair correlation with CD8 (Spearman r 0.53, *P* < 0.001) and CD4 (Spearman r 0.46, *P* < 0.001).

#### Association between TIME markers and 21-gene RS

**Fig. 2:**
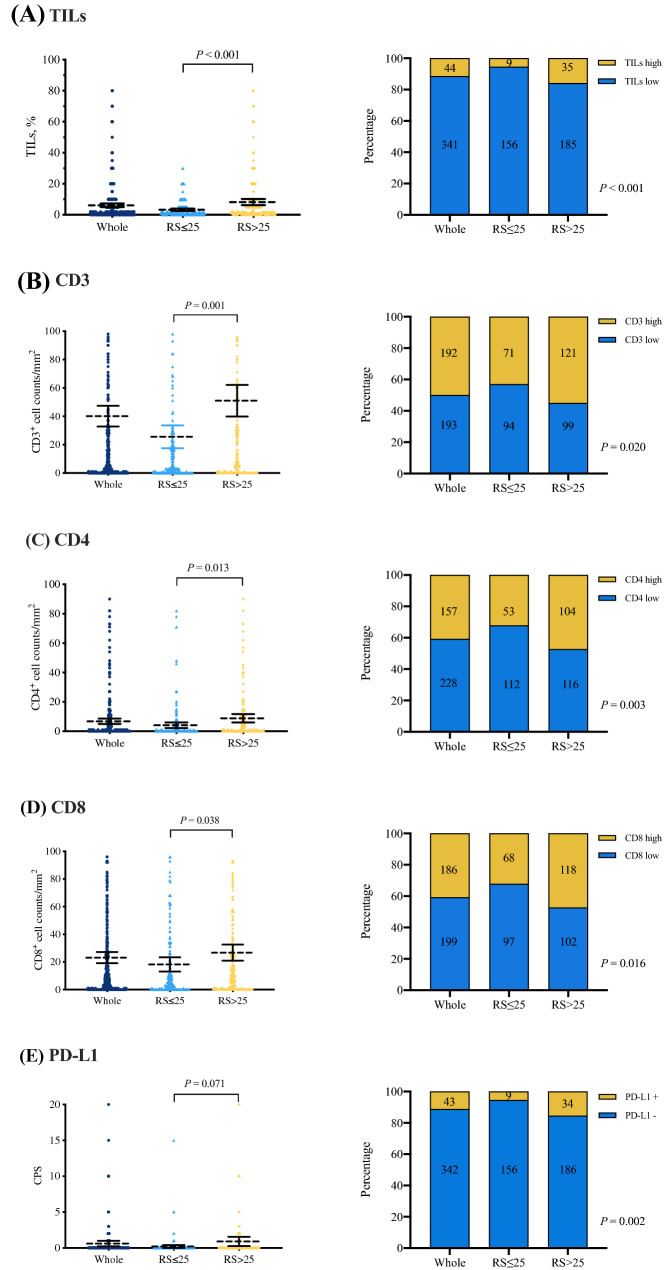
Association of TIME markers with RS. Counts of stromal TILs, CD3 + , CD4 + , CD8 + cells, and CPS were compared using Kruskal–Wallis test between RS categories. Dashed line represents average value with 95% confidence interval. Distribution of cases with high and low TIME markers were compared using Chi-square test between RS categories. *TIL* tumor-infiltrating lymphocyte, *RS* recurrence score, *PD-L1* programmed cell death-ligand 1, *CPS* combined positive score, *TIME* tumor immune microenvironment

Among the included population, 165 (42.9%) and 220 (57.1%) patients had RS ≤ 25 and RS > 25, respectively. The averages of the TIME markers were compared according to RS, and we found that those with RS > 25 had significantly higher average TILs (8.24% vs 3.26%, *P* < 0.001; Fig. 2A), higher average CD3 (51.01 vs 25.58, *P* = 0.001; Fig. 2B), higher average CD4 (8.80 *vs* 4.09, *P* = 0.013; Fig. 2C), and higher average CD8 (26.74 *vs* 18.23, *P* = 0.038; Fig. 2D). The average PD-L1 expression was comparable between RS subgroups (CPS 0.20 *vs* 0.91, *P* = 0.071; Fig. 2E). We then compared the percentage of cases expressing higher TIME marker by RS categories. RS > 25 patients were more likely to have high TILs (15.8% vs 5.5%, *P* < 0.001), high CD3 (55.0% vs 43.0%, *P* = 0.020), CD4 (47.3% vs 32.1%, *P* = 0.003), CD8 (53.6% vs 41.2%, *P* = 0.016) and positive PD-L1 (15.5% vs 5.5%, *P* = 0.002), compared to those with RS ≤ 25. Spearman correlation test demonstrated that continuous RS was positively, but weakly associated with continuous stromal TILs (r 0.24, *P* < 0.001), CD3 (r 0.15, *P* = 0.004), CD8 (r 0.10, *P* = 0.047), but not with CD4 (r 0.09, *P* = 0.078). Furthermore, immune inflamed cases reported higher RS (42.95 ± 20.35), compared to immune excluded (32.89 ± 20.02, *P* = 0.007), and immune desert (35.51 ± 23.83, *P* = 0.058) cases, while the latter two had comparable RS (*P* = 0.320).

#### Association of TIME markers with single gene expression in RS panel

The distribution of individual gene expression in the 21-gene RS panel was studied according to the above TIME markers (Additional file 2: Figure S2). Compared to patients with TILs > 10%, those with low TILs had significantly higher ER group genes including *Bcl2* (*P* = 0.002; Additional file 2: Figure S2A) and *CEGP1* (*P* = 0.003); lower proliferation group genes including *CCNB1* (*P* = 0.002), *Ki67* (*P* = 0.001), *MYBL2* (*P* = 0.006), *STK15* (*P* < 0.001), and *SURV* (*P* < 0.001); lower *CTSL2* (*P* = 0.006); as well as lower *BAG1* (*P* < 0.001) mRNA levels.

High CD3 was associated with lower *CEGP1* (*P* = 0.049; Additional file 2: Figure S2B), as well as higher proliferation-related genes including *CCNB1* (*P* = 0.032), *MYBL2* (*P* = 0.001), and *SURV* (*P* = 0.022). High CD4 was also related to lower *CEGP1* (*P* = 0.017; Additional file 2: Figure S2C), higher proliferation group genes *Ki67* (*P* = 0.001), *STK15* (*P* = 0.004), *SURV* (*P* = 0.001), as well as higher invasion group genes as *CTSL2* (*P* = 0.001) and *STMY3* (*P* = 0.013). Meanwhile, *CEGP1* (*P* = 0.044; Additional file 2: Figure S2D) was also adversely associated with CD8. When comparing single gene expression according to immune phenotype, we found that immune inflamed tumors had a significantly lower mRNA level of *CEGP1* (*P* = 0.009; Additional file 2: Figure S2E), higher *CTSL2* (*P* = 0.048), and lower *BAG1* (*P* = 0.004) than immune excluded and immune desert cases.

Moreover, PD-L1-positive patients tended to have lower ER group genes including *ER* (*P* = 0.017; Additional file 2: Figure S2F), *PR* (*P* = 0.017) and *CEGP1* (*P* < 0.001); higher proliferation group genes including *CCNB1* (*P* = 0.039), *Ki67* (*P* = 0.003), *MYBL2* (*P* = 0.037), *STK15* (*P* = 0.004), and *SURV* (*P* < 0.001); higher invasion group genes including *CTSL2* (*P* = 0.007) and *STMY3* (*P* = 0.003); as well as lower *GSTM1* (*P* = 0.001) mRNA levels.

#### Clinical outcomes according to TIME profiling

**Fig. 3:**
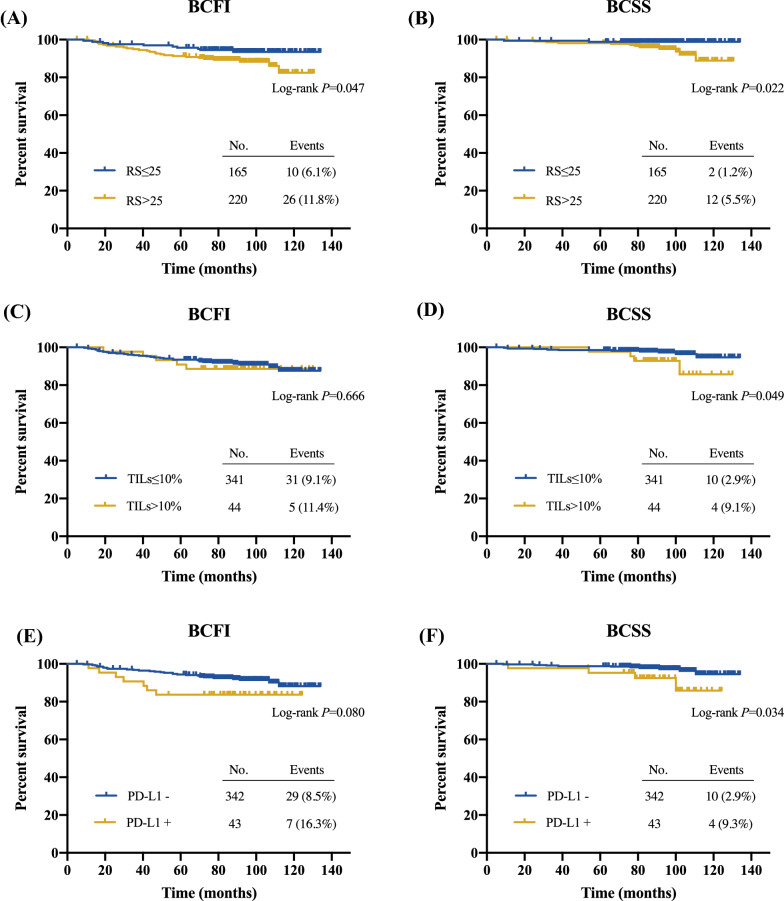
Clinical outcomes of Luminal-like patients according to RS, TILs, and PD-L1 level. BCFI and BCSS were compared by RS and TIME markers using Kaplan–Meier curves. *BCFI* breast cancer-free interval, *RS* recurrence score, *BCSS* breast cancer-specific survival, *TIL* tumor infiltrating lymphocyte, *PD-L1* programmed cell death-ligand 1

After a median follow-up of 91.67 (range 5.03–134.03) months, 36 (9.35%) breast cancer-related events were observed, including 5 local regional recurrences, 11 distant metastases, 6 contralateral breast cancer, and 14 breast cancer-specific deaths. RS > 25 was associated with significantly decreased 5-year BCFI (91.3% vs 95.7%, *P* = 0.047; Fig. 3A) and 5-year BCSS (98.2% vs 98.8%, *P* = 0.022; Fig. 3B).

Moreover, BCFI was compared according to patients’ immune profiles. In all, high TILs and low TILs subgroups reported similar BCFI (90.9% vs 93.5%, *P* = 0.666; Fig. 3C). Moreover, BCFI was comparable between patients with different levels of CD3, CD4, CD8, immune phenotype, or PD-L1 (all *P* > 0.05; Fig. 3E, Additional file 2: Figure S3). when further stratified by luminal subtype, we found that luminal B patients with low TILs and PD-L1 negative status tended to have better BCFI (*P* = 0.087, Additional file 2: Figure S4).

Regarding BCSS, those with low TILs had significantly superior 5-year BCSS compared to those with high TILs (98.5% vs 97.7%, *P* = 0.049; Fig. 3D). PD-L1 positive patients had an impaired 5-year BCSS than PD-L1-negative ones (98.8% vs 95.3%, *P* = 0.034; Fig. 3F). BCSS was not different between subgroups when divided by the other TIME markers (all *P* > 0.05; Additional file 2: Figure S3).

**Fig. 4:**
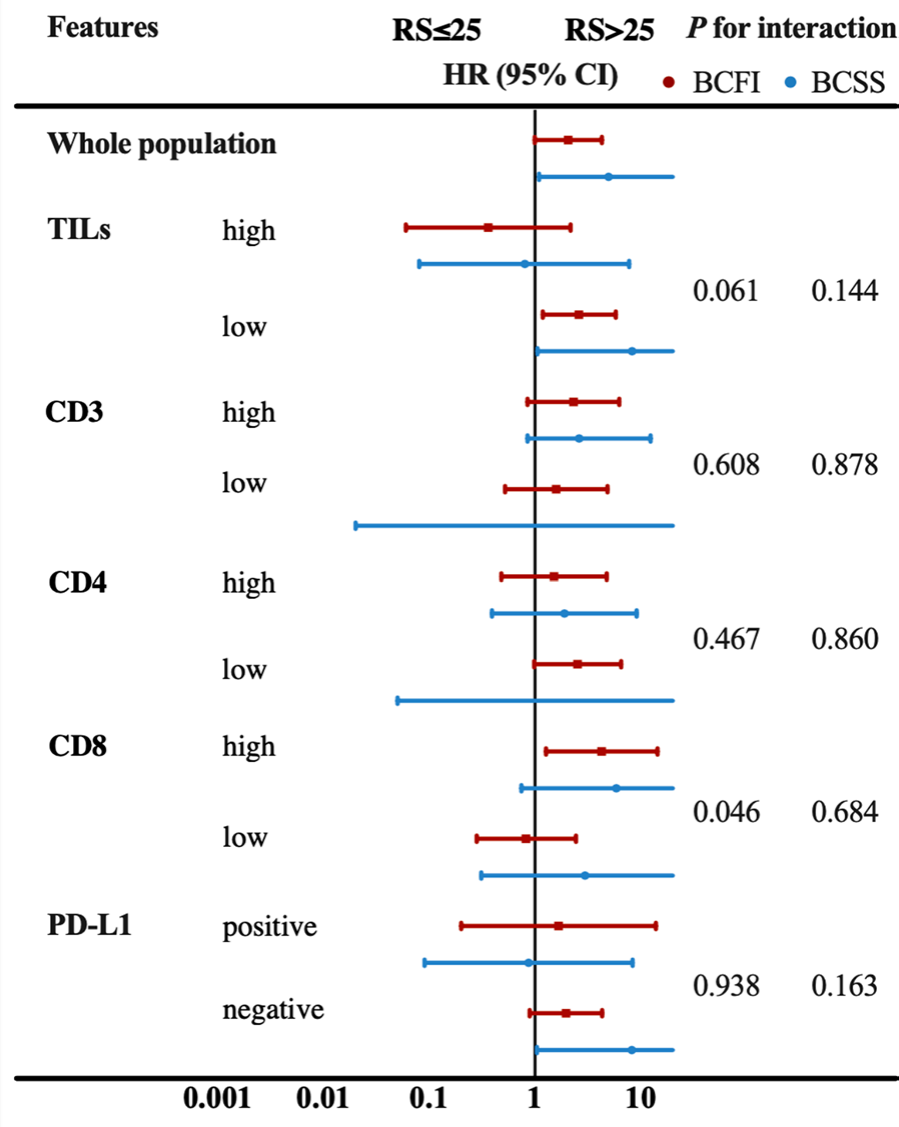
Interaction of RS and TIME markers on BCFI and BCSS. HR with 95% CI were compared between RS categories by each TIME subgroup. *RS* recurrence score, *HR* hazard ratio, *CI* confidence interval, *BCFI* breast cancer-free interval, *BCSS* breast cancer-specific survival, *TIL* tumor-infiltrating lymphocyte, *PD-L1* programmed cell death-ligand 1

Further subgroup analysis showed that 21-gene RS interacted with CD8 expression on BCFI (Fig. 4). For those with high CD8 expression, RS > 25 significantly increased the risk of BCFI (hazard ratio 4.27, 95% CI 1.27–14.43; Fig. 4), while such association was not found in those with weak CD8 (hazard ratio 0.82, 95% CI 0.28–2.44, *P* for interaction = 0.046). Other TIME markers did not interact with 21-gene RS on BCFI or BCSS (Fig. 4). Further likelihood ratio tests were conducted to test if TIME markers could add prognostic value on RS (dditional file 3: Table S7). We found that TILs tended to add prognostic value to RS in luminal A patients (*P* for BCSS = 0.083). CD8 tented to add prognostic value to RS in endocrine therapy-treated patients (*P* for BCFI = 0.077, *P* for BCSS = 0.083). CD3 tended to add prognostic value to RS in chemotherapy-treated patients (*P* for BCFI = 0.066, *P* for BCSS = 0.059). In addition, immune phenotype added significant prognostic value to RS in chemotherapy-treated patients (*P* for BCSS = 0.020).

## Discussion

Our study included Luminal breast cancer patients with 21-gene RS and TIME profiling and found that stromal TILs, CD3, CD4, CD8, and PD-L1 were generally positively correlated with each other. Continuous RS showed a weak correlation with continuous TILs, CD3, CD8, and PD-L1, but not with CD4. Regarding single gene expression in the 21-gene RS panel, higher immune infiltration was related to lower mRNA level of ER group genes, higher mRNA level of proliferation and invasion group genes. In terms of disease outcomes, TILs, and PD-L1 were inversely associated with BCSS, while the other TIME markers could not individually predict disease outcomes. To our knowledge, this is the largest study to explore the comprehensive association of TIME markers with 21-gene RS and the prognostic value of host immune profile in Luminal breast cancer patients, which can provide evidence on the mutual relationship between tumor genetic profile and tumor immune microenvironment.

Great heterogeneity exists in the tumor immune microenvironment. The quantity and quality of breast cancer immune cell infiltrate, including the presence of different lymphocyte subsets, are key to understanding the controversial results from immune profile studies. Therefore, CD3, CD4, CD8, as well as PD-L1 were selected as immune markers of interest in the current study. Our results suggested a positive association between each two of the TIME markers. This is generally in line with previous studies [15, 26–28]. Such result might be attributed to the mutual dependency of different subsets of immune cells in adaptive immunity to function [29]. However, unlike a strong correlation between immune markers in triple negative breast cancer [15], here the correlation was rather modest in the Luminal-like, HER2-negative population. This was possibly because most HR-positive tumors were less immunogenic than HR-negative ones, and the distribution of immune-related genes were disparate between molecular subtypes [30, 31].

Previous studies found that CD4^+^, CD8^+^ TILs infiltration was substantially higher in tumors with more aggressive tumor behaviors, such as high grade, negative HR status, HER2 amplification, and high Ki-67 level [32, 33]. Our data in HR-positive, HER2-negative, node-negative population confirmed that TILs expression was positively correlated with higher grade and higher Ki-67. However, we showed that the expression of different immune markers was influenced by distinct clinicopathologic factors. These findings reflected that the differently distributed immune cell subsets motivated and regulated by tumor-associated antigens might lead to heterogenous effect of immune infiltrate on tumors.

Few studies have investigated the association between 21-gene RS and TIME markers. A significant but weak correlation between continuous RS and continuous TILs was observed in a cohort of 198 ER-positive, HER2-negative patients (Pearson's *r* 0.201, *P* = 0.004) [34]. In the same study, Luminal-like patients presenting high TIL levels tended to have RS > 25, but the trend was not statistically significant (*P* = 0.155) [34]. Besides, a translational analysis of WSG Plan B trial identified TILs to be significantly and strongly correlated with RS categories in univariate model [33]. Consistently, we found that patients with RS > 25 had significantly higher average TILs. Another highlight of our study was that we also compared the expression of a single gene in the RS panel by immune cell expression levels and found that higher immune infiltration was consistently associated with lower ER group, higher proliferation group, higher invasion group genes mRNA level. To our knowledge this is the first study that comprehensively analyzes the association of RS with different immune markers including TILs, CD3, CD4, CD8, and PD-L1, as both continuous and categorical variables in Luminal-like population and also analyzed the single gene expression level in the RS panel. Our findings added to the notion that high immune infiltrates in HR-positive tumors may be more related to more aggressive tumor biology behavior.

The prognostic value of TILs was heterogeneous for different breast cancer molecular subtypes. A joint analysis of four adjuvant studies showed that TILs expression was associated with prognosis only in HER2-positive (*P* = 0.025) and triple negative (*P* < 0.001) subtypes, with no significant survival difference for HR-positive, HER2-negative population [10]. Data from WSG Plan B trial showed no significant difference in clinical outcomes among stromal TILs categories in HR-positive patients [33]. Moreover, as established by another pooled analysis, continuous TILs enumeration was not associated with disease-free survival (DFS), but significantly inversely associated with overall survival (OS) in Luminal-like, HER2-negative patients [11]. In consistence with these findings, results from our cohort demonstrated that TILs ≤ 10% subgroup had similar 5-year BCFI compared to those with TILs > 10% but had significantly improved 5-year BCSS compared to TILs > 10% subgroup. The adverse effect of TILs on survival of Luminal-like patients might be partially explained by the potential resistance to adjuvant endocrine therapy, since high lymphocytic infiltration as well as high expression of immune-related genes were shown to be related to poor response to anastrozole in a previous study [35]. Another possible mechanism might lie in the fact that dendritic cells could polarize CD4^+^ T cells, and lead to a poor response to estrogen deprivation [35]. Moreover, the diversity in the tumor microenvironment with distinct cancer-associated stromal cells among molecular subtypes also contributed to explaining the difference [36]. With a further identification and subtyping of Luminal-like tumors according to, for example, gene expression signatures, more information would be provided to understand tumor immunobiology.

In the current cohort, we established an interaction between RS and TIME markers on survival in HR-positive/HER2-negative patients. The prognostic value of RS tended to be more significant in CD8 high, and in TILs low patients. According to previous reports, Chan et al*.* established that the CD8 + /T_reg_ ratio was associated with better response to endocrine therapy [37], indicating high CD8 patients might be more endocrine-sensitive. Dieci et al*.* found that after neoadjuvant chemotherapy, a significant Ki67 suppression was observed in patients with low TILs (*P* = 0.001) but not in the high TILs group (*P* = 0.612) in HR + HER2- disease [38]. Meantime, we found that TILs, CD8, CD3 and immune phenotype tended to add prognostic value to RS different subgroups. Taken together, we suggested the interaction of RS and TIME markers on survival was further affected by luminal subtype, as well as the treatment that patients received, which warranted further exploration.

Apart from the strengths, several other key limitations need to be further investigated. First of all, given that Luminal-like breast cancer had much lower immune infiltration than other more aggressive subtypes, the standardization of immune marker enumeration and the selection of optimal cutoffs are mandatory for Luminal-like subtype. Second, with our center-specific RS assay, different RS cutoffs should be further tested to better understand the association between genetic and immunologic signatures. Meanwhile, here we used anti PD-L1 antibody clone 28–8 instead of 22C3, though previous study showed a highly comparable staining by the 22C3 and 28–8, our findings need to be validated with other standard antibodies [39]. Furthermore, a deeper clustering of immune cells with the help of novel technology, such as single-cell RNA sequencing [40], using more precise markers, for example, to identify Foxp3^+^ Treg cells from CD4^+^ T cells, and in different tumor regions, might provide further insight into the distinct prognostic role of various immune cell subsets in the tumor microenvironment.

In conclusion, stromal TILs, CD3, CD4, CD8 and tumor PD-L1 were generally positively correlated with each other in HR-positive/HER2-negative, breast cancer patients. Besides, higher TIME markers tended to be related to a higher 21-gene RS. Patients with higher TIME expression level had lower ER group, higher proliferation group, and higher invasion group genes mRNA levels in the 21-gene RS panel. TILs infiltration and PD-L1 were inversely associated with BCSS, which warranted further exploration.

## Supplementary Information

**Additional file 1: Appendix.** Quality control of the center-specific 21 gene RS assay.

**Additional file 2: Figure S1.** Study population flowchart. **Figure S2.** Single gene expression from 21-gene RS panel according to TIME markers. **Figure S3.** Clinical outcomes of Luminal-like patients according to TIME markers. **Figure S4.** BCFI according to RS, TILs, and PD-L1 level by luminal subtypes.

**Additional file 3: Table S1.** Adjuvant treatment. **Table S2.** Univariate analysis of clinicopathologic factors associated with TIME markers. **Table S3.** Multivariate analysis of clinicopathologic factors associated with TILs. **Table S4.** Multivariate analysis of clinicopathologic factors associated with CD3, CD4 and CD8. **Table S5.** Multivariate analysis of clinicopathologic factors associated with immune phenotype. **Table S6.** Multivariate analysis of clinicopathologic factors associated with PD-L1. **Table S7.** Likelihood ratio test of TIME markers on survival.

## Data Availability

The data sets analyzed during the current study are available from the corresponding authors on reasonable request.
