# Supplementary material for: Association of tumor immune microenvironment profiling and 21-gene recurrence assay in early breast cancer patients
Source: Eur J Med Res. 2022 Dec 17;27:293. doi: 10.1186/s40001-022-00917-3 (PMC9758791; doi:10.1186/s40001-022-00917-3)
Supplement: Supplementary file 1 — Additional file 1: Appendix. Quality control of the center-specific 21 gene RS assay. [file 40001_2022_917_MOESM1_ESM.docx]

**
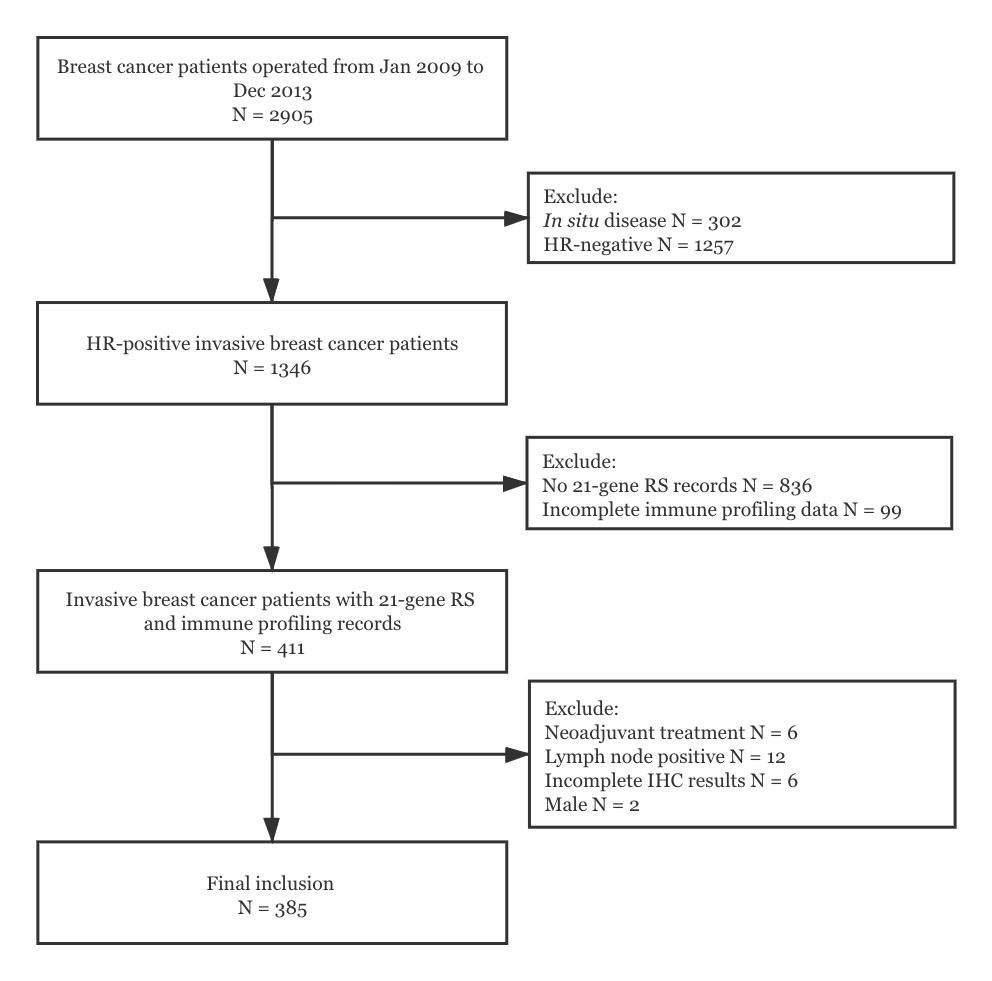
**

**Supplementary Figure S1. Study population flowchart.**

Abbreviations: HR, hormone receptor; RS, recurrence score; IHC, immunohistochemistry.

**Supplementary Figure S2. Single gene expression from 21-gene RS panel according to TIME markers.** The mRNA expression of genes in RS panel were tested by qRT-PCR assay, measured by cycle threshold, adjusted by endogenous genes β-actin, GAPDH, GUS, RPLPO, and TFRC, and recorded as ΔC_T_= C_T_ _Gene_ – C_T_ _Reference_. ΔC_T_ was compared using Kruskal-Wallis test to compare ΔC_T_ by different TIME markers. **P* <0.05, ** *P* <0.01, **** *P* <0.001, **** *P* <0.0001.

Abbreviations: TIL, tumor-infiltrating lymphocyte; C_T_, cycle threshold; HER2, human epidermal growth factor receptor-2; PD-L1, programmed cell death-ligand 1; RS, recurrence score; TIME, tumor immune microenvironment.

**Supplementary Figure S3. Clinical outcomes of Luminal-like patients according to TIME markers.** BCFI and BCSS were compared by TIME markers using Kaplan–Meier curves.

Abbreviations: BCFI, breast cancer-free interval; BCSS, breast cancer-specific survival.

**Supplementary Figure S4. BCFI according to RS, TILs, and PD-L1 level by luminal subtypes.**

Abbreviations: BCFI, breast cancer-free interval; RS, recurrence score; TIL, tumor infiltrating lymphocyte; PD-L1, programmed cell death-ligand 1.
