# Supplementary material for: Association of tumor immune microenvironment profiling and 21-gene recurrence assay in early breast cancer patients
Source: Eur J Med Res. 2022 Dec 17;27:293. doi: 10.1186/s40001-022-00917-3 (PMC9758791; doi:10.1186/s40001-022-00917-3)
Supplement: Supplementary file 2 — Additional file 2: Figure S1. Study population flowchart. Figure S2. Single gene expression from 21-gene RS panel according to TIME markers. Figure S3. Clinical outcomes of Luminal-like patients according to TIME markers. Figure S4. BCFI according to RS, TILs, and PD-L1 level by luminal subtypes. [file 40001_2022_917_MOESM2_ESM.docx]

**Supplementary Table S1. Adjuvant treatment.**

|  | All | RS≤25 | RS>25 |
| --- | --- | --- | --- |
| Chemotherapy |  |  |  |
| Yes | 209 | 77 | 132 |
| No | 176 | 88 | 88 |
| Radiation therapy |  |  |  |
| Yes | 130 | 57 | 73 |
| No | 255 | 108 | 147 |
| Endocrine therapy |  |  |  |
| Yes | 365 | 156 | 209 |
| No | 20 | 9 | 11 |

Abbreviations: RS, recurrence score.

**Supplementary Table S2. Univariate analysis of clinicopathologic factors associated with TIME markers**

| Characteristics | Univariate *P* value | | | | | |
| --- | --- | --- | --- | --- | --- | --- |
|  | TILs | CD3 | CD4 | CD8 | Immune Phenotype | PD-L1 |
| Age, years | 0.062 | 0.219 | 0.112 | 0.097 | **0.009** | **0.045** |
| Menstrual status | 0.400 | 0.130 | 0.258 | **0.028** | 0.118 | **0.048** |
| Histologic type | **0.029** | **0.004** | 0.594 | 0.681 | 0.852 | 0.213 |
| Tumor size, cm | 0.150 | 0.405 | **0.001** | 0.227 | **0.033** | 0.234 |
| Histological grade | **<0.001** | **0.024** | **0.011** | 0.632 | 0.177 | **0.015** |
| ER expression, % | **0.049** | 0.363 | 0.081 | 0.833 | **0.028** | **0.038** |
| PR status | **0.018** | 0.360 | 0.250 | 0.352 | 0.813 | **<0.001** |
| Ki-67 index, % | **<0.001** | **0.022** | **0.037** | 0.279 | **0.009** | **0.001** |
| Recurrence score | **0.001** | **0.020** | **0.003** | **0.016** | **0.008** | **0.002** |

Abbreviations: TIME, tumor immune microenvironment; TIL, tumor-infiltrating lymphocyte; PD-L1, programmed cell death-ligand 1; IDC, invasive ductal carcinoma; ER, estrogen receptor; PR, progesterone receptor.

**Supplementary Table S3. Multivariate analysis of clinicopathologic factors associated with TILs**

| Characteristics | TILs, % ($>$10 *vs* $\leq$10) | | |
| --- | --- | --- | --- |
|  | OR | 95% CI | *P* |
| Histologic type (Non-IDC *vs* IDC) | 0.22 | 0.03-1.78 | 0.155 |
| Histological grade |  |  | **0.025** |
| I *vs* III | 1.38 | 0.22-8.71 | 0.730 |
| II *vs* III | 0.39 | 0.18-0.82 | **0.013** |
| ER expression, % ($\geq$50 *vs* $<$50) | 0.56 | 0.25-1.26 | 0.162 |
| PR status (Negative *vs* Positive) | 2.07 | 0.85-5.00 | 0.108 |
| Ki-67 index, % ($\geq$14 *vs* $<$14) | 8.57 | 1.75-41.93 | **0.008** |
| RS (>25 *vs* ≤25) | 2.30 | 0.91-5.83 | 0.080 |

Abbreviations: TIL, tumor-infiltrating lymphocyte; OR, odds ratio; CI, confidence interval; IDC, invasive ductal carcinoma; ER, estrogen receptor; PR, progesterone receptor; Lum, Luminal; RS, recurrence score.

**Supplementary Table S4. Multivariate analysis of clinicopathologic factors associated with CD3, CD4 and CD8**

|  | CD3 (High *vs* Low) | | |  | CD4 (High *vs* Low) | | |  | CD8 (High *vs* Low) | | |
| --- | --- | --- | --- | --- | --- | --- | --- | --- | --- | --- | --- |
|  | OR | 95% CI | *P* |  | OR | 95% CI | *P* |  | OR | 95% CI | *P* |
| Menstruation (Pre- *vs* Post-) |  |  | NS |  |  |  | NS |  | 1.59 | 1.05-2.44 | **0.028** |
| Histologic type (IDC *vs* Non-IDC) | 0.43 | 0.22-0.83 | **0.012** |  |  |  | NS |  |  |  | NS |
| Tumor size, cm ($\leq$2 *vs* $>$2) |  |  | NS |  | 1.79 | 1.11-2.88 | **0.017** |  |  |  | NS |
| Histological grade |  |  | 0.258 |  |  |  | 0.089 |  |  |  | NS |
| I *vs* III | 1.08 | 0.45-2.61 | 0.857 |  | 0.40 | 0.16-0.99 | **0.047** |  |  |  | NS |
| II *vs* III | 0.72 | 0.43-1.20 | 0.203 |  | 0.97 | 0.59-1.62 | 0.920 |  |  |  | NS |
| Ki-67 index, % ($\geq$14 *vs* $<$14) | 1.38 | 0.78-2.44 | 0.273 |  | 0.87 | 0.49-1.56 | 0.639 |  |  |  | NS |
| RS (>25 *vs* ≤25) | 1.47 | 0.94-2.30 | 0.091 |  | 1.52 | 0.95-2.43 | 0.079 |  | 1.66 | 1.10-2.50 | **0.016** |

Abbreviations: OR, odds ratio; CI, confidence interval; IDC, invasive ductal carcinoma; NS, not significant; RS, recurrence score.

**Supplementary Table S5. Multivariate analysis of clinicopathologic factors associated with immune phenotype ^a^**

|  | Immune inflamed | | |  | Immune excluded | | | *P* |
| --- | --- | --- | --- | --- | --- | --- | --- | --- |
|  | OR | 95% CI | *P* |  | OR | 95% CI | *P* |  |
| Age, years ($<$50 *vs* $\geq$50) | 1.97 | 0.97-3.98 | 0.061 |  | 1.74 | 1.06-2.85 | **0.029** | **0.036** |
| Tumor size, cm ($\leq$2 *vs* $>$2) | 0.75 | 0.35-1.60 | 0.457 |  | 0.61 | 0.36-1.03 | 0.062 | 0.172 |
| ER, % ($<$50 *vs* $\geq$50) | 1.93 | 0.90-4.12 | 0.091 |  | 0.67 | 0.36-1.25 | 0.209 | 0.052 |
| Ki-67 index, % ($<$14 *vs* $\geq$14) | 0.45 | 0.22-0.96 | **0.039** |  | 0.83 | 0.51-1.35 | 0.455 | 0.100 |
| RS (>25 *vs* ≤25) | 0.38 | 0.17-0.88 | **0.023** |  | 0.94 | 0.58-1.54 | 0.812 | 0.057 |

^a^ The reference category was immune desert phenotype.

Abbreviations: OR, odds ratio; CI, confidence interval; ER, estrogen receptor; RS, recurrence score.

**Supplementary Table S6. Multivariate analysis of clinicopathologic factors associated with PD-L1**

|  | PD-L1 (Positive *vs* Negative) | | |
| --- | --- | --- | --- |
|  | OR | 95% CI | *P* |
| Age, years ($\leq$50 *vs* $>$50) | 2.03 | 0.49-8.37 | 0.327 |
| Menstruation (Post- *vs* Pre-) | 0.85 | 0.21-3.45 | 0.816 |
| Histological grade |  |  | 0.415 |
| I *vs* III | 1.04 | 0.20-5.35 | 0.967 |
| II *vs* III | 0.63 | 0.30-1.35 | 0.235 |
| ER, % ($\geq$50 *vs* $<$50) | 0.75 | 0.34-1.65 | 0.472 |
| PR status (Negative *vs* Positive) | 2.80 | 1.20-6.57 | **0.029** |
| Ki-67 index, % ($\geq$14 *vs* $<$14) | 2.68 | 0.82-8.74 | 0.745 |
| RS (>25 *vs* ≤25) | 2.36 | 0.85-5.61 | 0.052 |

Abbreviations: PD-L1, programmed cell death-ligand 1; OR, odds ratio; CI, confidence interval; ER, estrogen receptor; PR, progesterone receptor; Lum, Luminal; RS, recurrence score.

**Supplementary Table S7.** Likelihood ratio test of TIME markers on survival.

| TIME marker | BCFI | |  | BCSS | |
| --- | --- | --- | --- | --- | --- |
|  | △LR-χ2 | *P* |  | △LR-χ2 | *P* |
| All |  |  |  |  |  |
| RS | Reference |  |  | Reference |  |
| RS + CD3 | 1.89 | 0.167 |  | 2.04 | 0.153 |
| RS + CD4 | 0.52 | 0.472 |  | 0.13 | 0.719 |
| RS + CD8 | 1.94 | 0.164 |  | 1.67 | 0.197 |
| RS + TILs | 0.01 | 0.940 |  | 1.69 | 0.194 |
| RS + Immune phenotype | 0.21 | 0.644 |  | 0.99 | 0.319 |
| RS + PD-L1 | 1.66 | 0.197 |  | 2.10 | 0.148 |
| Luminal A |  |  |  |  |  |
| RS | Reference |  |  | Reference |  |
| RS + CD3* | 1.39 | 0.239 |  | / | / |
| RS + CD4* | 0.72 | 0.397 |  | / | / |
| RS + CD8 | 0.03 | 0.860 |  | <0.001 | 0.982 |
| RS + TILs | 0.70 | 0.403 |  | 3.01 | 0.083 |
| RS + Immune phenotype | 1.02 | 0.313 |  | 0.03 | 0.860 |
| RS + PD-L1* | / | / |  | / | / |
| Luminal B |  |  |  |  |  |
| RS | Reference |  |  | Reference |  |
| RS + CD3 | 1.01 | 0.315 |  | 0.70 | 0.402 |
| RS + CD4 | 0.33 | 0.563 |  | 0.43 | 0.512 |
| RS + CD8 | 2.01 | 0.156 |  | 1.71 | 0.191 |
| RS + TILs | 0.07 | 0.797 |  | 0.49 | 0.483 |
| RS + Immune phenotype | 1.02 | 0.311 |  | 1.42 | 0.234 |
| RS + PD-L1 | 2.05 | 0.153 |  | 2.06 | 0.151 |

Abbreviations: TIME, tumor immune microenvironment; LR, likelihood ratio; BCFI, breast cancer-free interval; BCSS, breast cancer-specific survival; RS, recurrence score; TILs, tumor infiltrating lymphocytes; PD-L1, programmed cell death-ligand 1.

*: too few events to carry out subgroup analysis.
