# Supplementary material for: Association of tumor immune microenvironment profiling and 21-gene recurrence assay in early breast cancer patients
Source: Eur J Med Res. 2022 Dec 17;27:293. doi: 10.1186/s40001-022-00917-3 (PMC9758791; doi:10.1186/s40001-022-00917-3)
Supplement: Supplementary file 3 — Additional file 3: Table S1. Adjuvant treatment. Table S2. Univariate analysis of clinicopathologic factors associated with TIME markers. Table S3. Multivariate analysis of clinicopathologic factors associated with TILs. Table S4. Multivariate analysis of clinicopathologic factors associated with CD3, CD4 and CD8. Table S5. Multivariate analysis of clinicopathologic factors associated with immune phenotype. Table S6. Multivariate analysis of clinicopathologic factors associated with PD-L1. Table S7. Likelihood ratio test of TIME markers on survival. [file 40001_2022_917_MOESM3_ESM.docx]

**Appendix. Quality control of the center-specific 21 gene RS assay**

1. **Panel primer list**

Primer and probe sequences applied for RS assay were listed in Supplemental Data Table 1.

**Supplemental Data Table 1.** Primer and probe sequences for RS assay.

| **Gene** | **Primer** | **Oligo sequence** |
| --- | --- | --- |
| **ACTB** | S0034/B-acti.f2 | CAGCAGATGTGGATCAGCAAG |
| **ACTB** | S4730/B-acti.p2 | CAGCAGATGTGGATCAGCAAG |
| **ACTB** | S0036/B-acti.r2 | AGGAGTATGACGAGTCCGGCCCC |
| **BAG1** | S1386/BAG1.f2 | GCATTTGCGGTGGACGAT |
| **BAG1** | S1387/BAG1.r2 | CGTTGTCAGCACTTGGAATACAA |
| **BAG1** | S4731/BAG1.p2 | GTTCAACCTCTTCCTGTGGACTGT |
| **BCL2** | S0043/Bcl2.f2 | CCCAATTAACATGACCCGGCAACCAT |
| **BCL2** | S4732/Bcl2.p2 | CAGATGGACCTAGTACCCACTGAGA |
| **BCL2** | S0045/Bcl2.r2 | TTCCACGCCGAAGGACAGCGAT |
| **CCNB1** | S1720/CCNB1.f2 | CCTATGATTTAAGGGCATTTTTCC |
| **CCNB1** | S1721/CCNB1.r2 | TTCAGGTTGTTGCAGGAGAC |
| **CCNB1** | S4733/CCNB1.p2 | CATCTTCTTGGGCACACAAT |
| **CD68** | S0067/CD68.f2 | TGTCTCCATTATTGATCGGTTCATGCA |
| **CD68** | S4734/CD68.p2 | TGGTTCCCAGCCCTGTGT |
| **CD68** | S0069/CD68.r2 | CTCCAAGCCCAGATTCAGATTCGAGTCA |
| **SCUBE2** | S1494/CEGP1.f2 | CTCCTCCACCCTGGGTTGT |
| **SCUBE2** | S1495/CEGP1.r2 | TGACAATCAGCACACCTGCAT |
| **SCUBE2** | S4735/CEGP1.p2 | TGTGACTACAGCCGTGATCCTTA |
| **CTSL2** | S4354/CTSL2.f1 | CAGGCCCTCTTCCGAGCGGT |
| **CTSL2** | S4355/CTSL2.r1 | TGTCTCACTGAGCGAGCAGAA |
| **CTSL2** | S4356/CTSL2.p1 | ACCATTGCAGCCCTGATTG |
| **ESR1** | S0115/EstR1.f1 | CTTGAGGACGCGAACAGTCCACCA |
| **ESR1** | S4737/EstR1.p1 | CGTGGTGCCCCTCTATGAC |
| **ESR1** | S0117/EstR1.r1 | CTGGAGATGCTGGACGCCC |
| **GAPD** | S4738/GAPDH.p1 | GGCTAGTGGGCGCATGTAG |
| **GAPD** | S0374/GAPDH.f1 | CCGTTCTCAGCCTTGACGGTGC |
| **GAPD** | S0375/GAPDH.r1 | ATTCCACCCATGGCAAATTC |
| **GRB7** | S0130/GRB7.f2 | GATGGGATTTCCATTGATGACA |
| **GRB7** | S4726/GRB7.p2 | CCATCTGCATCCATCTTGTT |
| **GRB7** | S0132/GRB7.r2 | CTCCCCACCCTTGAGAAGTGCCT |
| **GSTM1** | S2026/GSTM1.r1 | GGCCACCAGGGTATTATCTG |
| **GSTM1** | S2027/GSTM1.f1 | GGCCCAGCTTGAATTTTTCA |
| **GSTM1** | S4739/GSTM1.p1 | AAGCTATGAGGAAAAGAAGTACACGAT |
| **GUSB** | S0139/GUS.f1 | TCAGCCACTGGCTTCTGTCATAATCAGGAG |
| **GUSB** | S4740/GUS.p1 | CCCACTCAGTAGCCAAGTCA |
| **GUSB** | S0141/GUS.r1 | TCAAGTAAACGGGCTGTTTTCCAAACA |
| **ERBB2** | S0142/HER2.f3 | CACGCAGGTGGTATCAGTCT |
| **ERBB2** | S4729/HER2.p3 | CGGTGTGAGAAGTGCAGCAA |
| **ERBB2** | S0144/HER2.r3 | CCAGACCATAGCACACTCGGGCAC |
| **MKI67** | S4741/Ki-67.p2 | CCTCTCGCAAGTGCTCCAT |
| **MKI67** | S0436/Ki-67.f2 | CCACTTGTCGAACCACCGCTCGT |
| **MKI67** | S0437/Ki-67.r2 | CGGACTTTGGGTGCGACTT |
| **MYBL2** | S3270/MYBL2.f1 | TTACAACTCTTCCACTGGGACGAT |
| **MYBL2** | S3271/MYBL2.r1 | GCCGAGATCGCCAAGATG |
| **MYBL2** | S4742/MYBL2.p1 | CTTTTGATGGTAGAGTTCCAGTGATTC |
| **PGR** | S1336/PR.f6 | GCATCAGGCTGTCATTATGG |
| **PGR** | S1337/PR.r6 | AGTAGTTGTGCTGCCCTTCC |
| **PGR** | S4743/PR.p6 | TGTCCTTACCTGTGGGAGCTGTAAGGTC |
| **RPLP0** | S0256/RPLP0.f2 | CCATTCTATCATCAACGGGTACAA |
| **RPLP0** | S4744/RPLP0.p2 | TCTCCACAGACAAGGCCAGGACTCG |
| **RPLP0** | S0258/RPLP0.r2 | TCAGCAAGTGGGAAGGTGTAATC |
| **AURKA** | S0794/STK15.f2 | CATCTTCCAGGAGGACCACT |
| **AURKA** | S0795/STK15.r2 | TCCGACCTTCAATCATTTCA |
| **AURKA** | S4745/STK15.p2 | CTCTGTGGCACCCTGGACTACCTG |
| **MMP11** | S2067/STMY3.f3 | CCTGGAGGCTGCAACATACC |
| **MMP11** | S2068/STMY3.r3 | TACAATGGCTTTGGAGGATAGCA |
| **MMP11** | S4746/STMY3.p3 | ATCCTCCTGAAGCCCTTTTCGCAGC |
| **BIRC5** | S0259/SURV.f2 | TGTTTTGATTCCCGGGCTTA |
| **BIRC5** | S4747/SURV.p2 | TGCCTTCTTCCTCCCTCACTTCTCACCT |
| **BIRC5** | S0261/SURV.r2 | CAAAGCTGTCAGCTCTAGCAAAAG |
| **TFRC** | S1352/TFRC.f3 | GCCAACTGCTTTCATTTGTG |
| **TFRC** | S1353/TFRC.r3 | ACTCAGGCCCATTTCCTTTA |
| **TFRC** | S4748/TFRC.p3 | AGGGATCTGAACCAATACAGAGCAGACA |

1. **Quality control**

Samples were tested by the same manipulator at different time points and by different manipulators following the same procedure. Intraclass correlation coefficient with 95% confidence interval was adopted to evaluate the consistency and reproducibility of the assay (Supplemental Data Table 2).

**Supplemental Data Table 2.** ICC of the RS genes.

|  | **Same manipulator** | | **Different manipulators** | |
| --- | --- | --- | --- | --- |
|  | **ICC** | **95% CI** | **ICC** | **95% CI** |
| **GRB7** | 0.846 | 0.620，0.935 | 0.824 | 0.678，0.904 |
| **HER2** | 0.844 | 0.652，0.931 | 0.743 | 0.531，0.859 |
| **ER** | 0.816 | 0.589，0.918 | 0.887 | 0.794，0.938 |
| **PR** | 0.934 | 0.829，0.972 | 0.896 | 0.766，0.949 |
| **Bcl2** | 0.807 | 0.558，0.915 | 0.797 | 0.629，0.889 |
| **CEGP1** | 0.980 | 0.955，0.991 | 0.955 | 0.918，0.976 |
| **CCNB1** | 0.776 | 0.486，0.902 | 0.598 | 0.275，0.778 |
| **Ki67** | 0.754 | 0.442，0.892 | 0.827 | 0.683，0.906 |
| **MYBL2** | 0.831 | 0.622，0.925 | 0.789 | 0.612，0.885 |
| **STK15** | 0.879 | 0.725，0.947 | 0.771 | 0.579，0.875 |
| **SURV** | 0.867 | 0.699，0.941 | 0.822 | 0.676，0.903 |
| **CTSL2** | 0.604 | 0.136，0.822 | 0.422 | -0.071，0.687 |
| **STMY3** | 0.844 | 0.644，0.931 | 0.783 | 0.602，0.881 |
| **CD68** | 0.097 | -1.150，0.610 | 0.668 | 0.396，0.818 |
| **GSTM1** | 0.851 | 0.660，0.935 | 0.708 | 0.463，0.841 |
| **BAG1** | 0.953 | 0.893，0.979 | 0.700 | 0.448，0.837 |
| **RS** | 0.984 | 0.964，0.993 | 0.986 | 0.974，0.992 |

Abbreviations: ICC, intraclass correlation coefficient; CI, confidence interval; RS, recurrence score.

1. **Concordance of RT-PCR with immunohistochemistry**

The RT-PCR results of ER and PR were judged according to the following criteria: ER score ≥ 6.5 was ER positive, < 6.5 was ER negative, PR score ≥ 5.5 was PR positive, and < 5.5 was PR negative. Compared with IHC results, RT-PCR results showed a high concordance rate of 98.7% for ER, and 87.8% for PR (Supplemental Data Table 3, Supplemental Data Figure 1).

**Supplemental Data Table 3. Concordance rate between RT-PCR and IHC for ER and PR.**

| **RT-PCR** | **IHC (case)** | | | **Concordance rate (%)** |
| --- | --- | --- | --- | --- |
|  | **Positive** | **Negative** | **Total** |  |
| ER |  |  |  | 98.7 |
| Positive | 915 | 0 | 915 |  |
| Negative | 12 | 0 | 12 |  |
| Total | 927 | 0 | 927 |  |
| PR |  |  |  | 87.8 |
| Positive | 665 | 67 | 732 |  |
| Negative | 36 | 74 | 110 |  |
| Total | 701 | 141 | 842 |  |

Abbreviations: RT-PCR, reverse transcription polymerase chain reaction; IHC, immunohistochemistry; ER, estrogen receptor; PR, progesterone receptor.


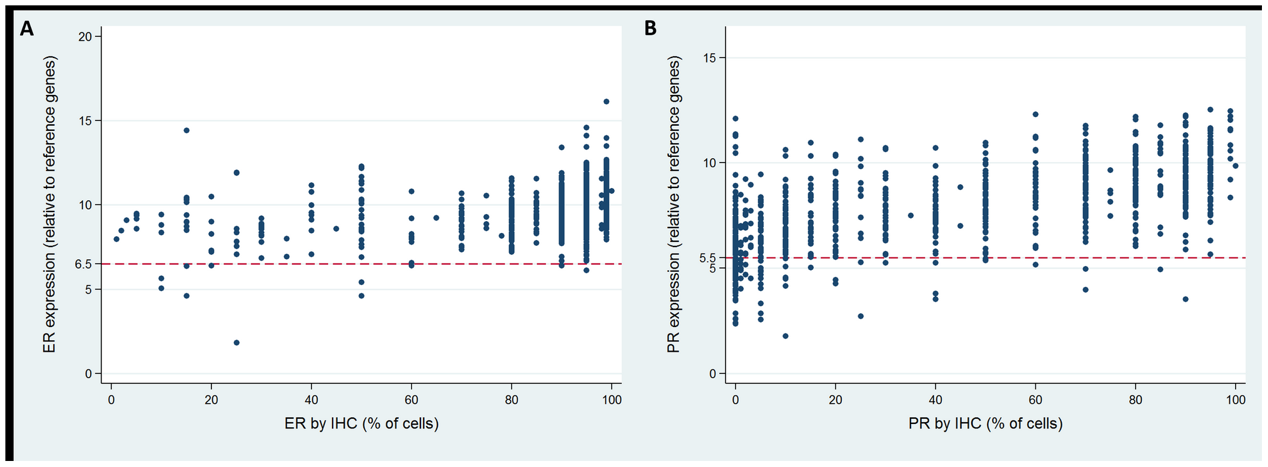


**Supplemental Data Figure 1.** Concordance of estrogen receptor (ER) and progesterone receptor (PR) status between RT-PCR and IHC. (A) ER; (B) PR.

Previous published data from Wu J, et al. Oncotarget. 2017;8(24):38706-38716.

1. **Prognostic and predictive value of RS**

Kaplan-Meier survival analysis found that the 4-year IDFS rates of patients in the low-risk, intermediate-risk and high-risk groups were 98.7%, 96.0% and 91.8%, respectively. There was a statistically significant difference in IDFS among the three groups (*P*=0.027), with the IDFS of the patients in the risk group significantly better than that of the patients in the high-risk group (*P*=0.018).

The 4-year IDFS rates of the low-risk, medium-risk, and high-risk groups of RS in node-negative patients were 98.7%, 95.8%, and 91.6%, respectively, and there was a statistically significant difference (*P*=0.027, Supplemental Data Figure 2). The IDFS of the low-risk group was significantly better than that of the high-risk group (*P*=0.017).

For node-positive patients, low risk RS was also significantly associated with better IDFS (hazard ratio 2.45, 95% CI 1.017-5.902, *P* = 0.046) compared to high risk ones.

In addition, we also tested the predictive value of RS. There were significant differences in the proportion of RS low-risk, intermediate-risk, and high-risk patients receiving chemotherapy, which were 17.1% (33/193), 56.4% (229/406), and 91.8% (123/134), respectively (*P* < 0.001, Supplemental Data Figure 3). Compared with the RS low risk group, a higher proportion of patients in the RS intermediate risk and high risk groups received chemotherapy. This trend in Luminal A and Luminal B patients was consistent with that in the general population. The proportions of RS low-risk, intermediate-risk, and high-risk patients undergoing chemotherapy were 6.5% (6/93), 29.9% (38/127), and 90.9% (10/11) (*P* < 0.001) and 27.0% (27/100), 68.5% (191/279), 91.9% (113/123) (*P* < 0.001).


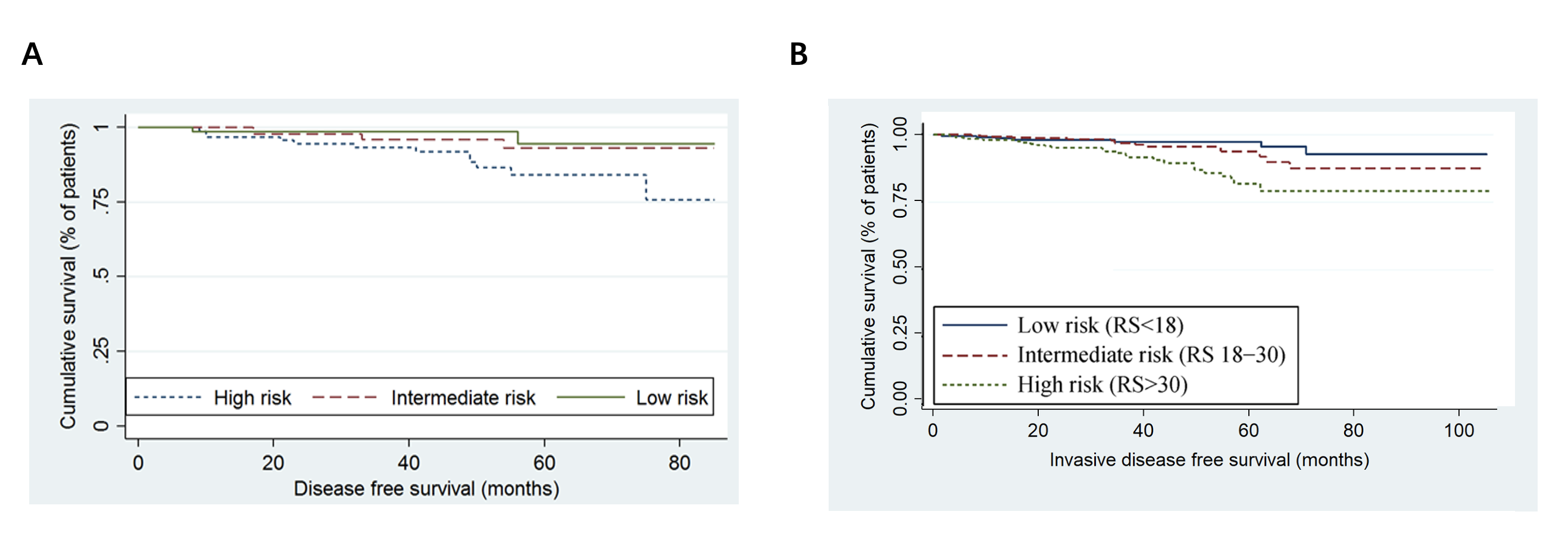


**Supplemental Data Figure 2.** Kaplan–Meier analysis according to RS categories in (A) node-negative and (B) node-positive patients.

Abbreviations: RS, recurrence score.

Previous published data from Wu J, et al. Front Med. 2020. doi: 10.1007/s11684-020-0738-0.


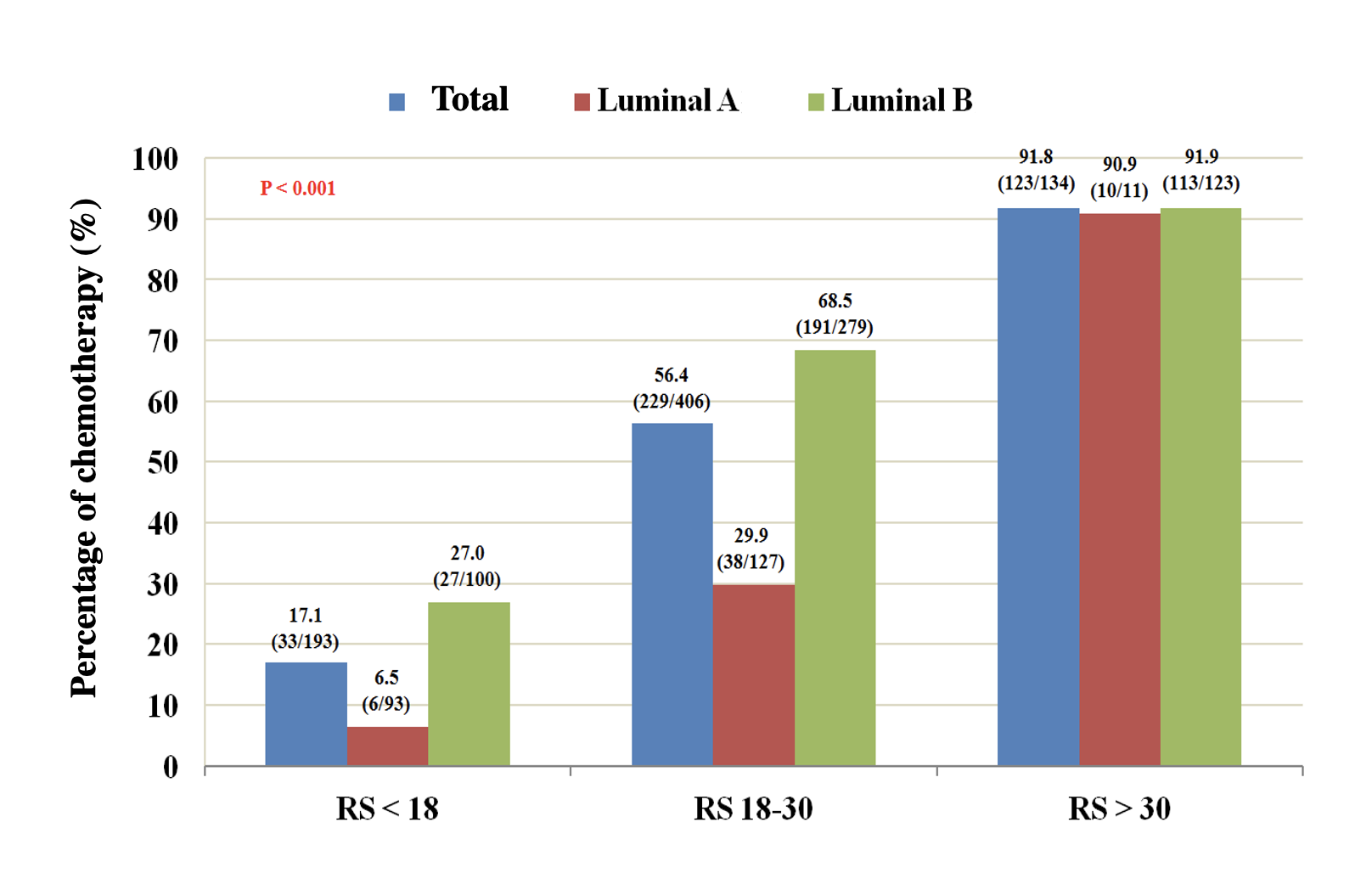


**Supplemental Data Figure 3.** Percentage of chemotherapy in patients with different RS results.

Abbreviations: RS, recurrence score.
